# Microbial Electrosynthesis Inoculated with Anaerobic Granular Sludge and Carbon Cloth Electrodes Functionalized with Copper Nanoparticles for Conversion of CO_2_ to CH_4_

**DOI:** 10.3390/nano12142472

**Published:** 2022-07-19

**Authors:** Sofia Georgiou, Loukas Koutsokeras, Marios Constantinou, Rafał Majzer, Justyna Markiewicz, Marcin Siedlecki, Ioannis Vyrides, Georgios Constantinides

**Affiliations:** 1 Department of Chemical Engineering, Cyprus University of Technology, 57 Anexartisias Str., P.O. Box 50329, Limassol 3603, Cyprus; sog.georgiou@gmail.com; 2 Department of Mechanical Engineering and Materials Science and Engineering, Cyprus University of Technology, Kitiou Kyprianou 45, Limassol 3041, Cyprus; l.koutsokeras@cut.ac.cy (L.K.); m.k.constantinou@cut.ac.cy (M.C.); 3 Research Unit for Nanostructured Materials Systems, Cyprus University of Technology, Kitiou Kyprianou 36, Limassol 3041, Cyprus; 4 Research and Innovation Centre Pro-Akademia, Innowacyjna 9/11, 95-050 Konstantynów Łódzki, Poland; rafal.majzer@proakademia.eu (R.M.); justyna.markiewicz@proakademia.eu (J.M.); marcin.siedlecki@proakademia.eu (M.S.)

**Keywords:** anaerobic granular sludge, microbial electrosynthesis, carbon cloth electrodes, copper nanoparticles

## Abstract

Microbial electrosynthesis (MES) can sustainably convert CO_2_ to products and significant research is currently being conducted towards this end, mainly in laboratory-scale studies. The high-cost ion exchange membrane, however, is one of the main reasons hindering the industrialization of MES. This study investigates the conversion of CO_2_ (as a sole external carbon source) to CH_4_ using membraneless MES inoculated with anaerobic granular sludge. Three types of electrodes were tested: carbon cloth (CC) and CC functionalized with Cu NPs, where Cu NPs were deposited for 15 and 45 min, respectively. During the MES experiment, which lasted for 144 days (six cycles), methane was consistently higher in the serum bottles with CC electrodes and applied voltage. The highest CH_4_ (around 46%) was found in the second cycle after 16 days. The system’s performance declined during the following cycles; nevertheless, the CH_4_ composition was twice as high compared to the serum bottles without voltage. The MES with Cu NPs functionalized CC electrodes had a higher performance than the MES with plain CC electrodes. Microbial profile analysis showed that the *Methanobacterium* was the most dominant genus in all samples and it was found in higher abundance on the cathodes, followed by the anodes, and then in the suspended biomass. The genus *Geobacter* was identified only on the anodes regarding relative bacterial abundance at around 6–10%. *Desulfovibrio* was the most dominant genus in the cathodes; however, its relative abundance was significantly higher for the cathodes with Cu NPs.

## 1. Introduction

Carbon dioxide (CO_2_) seriously affects the environment by contributing to the rise in the Earth’s surface temperature; 2020 was one of the hottest years in recorded history and extreme weather events occurred more frequently [1]. International treaties such as the Paris Agreement (with 196 signatories) show anthropogenic climate change as a worldwide public concern. Nations agreed on carbon trading and taxation as one way to view the economics of emissions reduction and society anticipates solutions by scientists and engineers with regards to CO_2_ emissions. However, CO_2_ is in a high oxidation state (+4) and is considered stable, so highly reducing conditions are required for its reduction. Several promising technologies have already been proposed that are mainly based on physicochemical methods but require extreme conditions, high energy, or expensive materials [2].

A generally cost-effective and environmentally friendly route for reducing CO_2_, by converting it into other useful organics, is microbial electrosynthesis (MES) [3,4]. MES utilizes anaerobic microbes as biocatalysts for the electricity-driven conversion of CO_2_ into products such as methane or volatile fatty acids (VFAs) under mild conditions [5]. The conversion of CO_2_ into CH_4_ takes place at the cathode via a biocatalytic reaction that involves electrochemically active microorganisms. The principles of MES in CO_2_-capturing and conversion have been outlined in several recent reviews [6,7]. In most studies for the conversion of CO_2_ to CH_4_, the MES systems used double chamber geometries, with the anode and cathode compartments separated by either an anion or cation exchange membrane. The membrane allows separate optimization of the cathode-catholyte and anode-anolyte. The cathode chamber can be inoculated with anaerobic pure chemolithoautotrophic bacteria or enriched with mixed methanogens or homoacetogens. In contrast, the anode chamber is either inoculated with unspecific exoelectrogens or maintained abiotic. The ion exchange membrane prevents oxygen produced at an aerobic anode from diffusing into an anaerobic cathode chamber and inhibits the anaerobic microorganisms in the cathode chamber [8].

In optimizing electromethanogenesis, reducing its cost, and potentially scaling-up the process, several reactor modification attempts have been employed over the years, the most notable of which relate to the removal of the ion exchange membrane and the modification of the used electrodes. The ion exchange membrane is in fact one of the main reasons hindering the industrialization of this process due to its high cost. It creates several technical challenges such as lowered mass transfer rate associated with membrane fouling, increased internal resistance and considerable voltage losses [8,9]. In addition, the ion exchange membrane contributes to a relatively large distance between electrodes in a double-chambered reactor which is another cause of higher ohmic losses [5,8]. Considering the total cost of reactor fabrication, ≈50–60% of the cost is due to the membrane and membrane maintenance cost [10]. A membraneless MES reactor, in which the CO_2_-containing gas sparged from the bottom of the reactor such as to prevent oxygen diffusion from the anode towards the cathode, was examined by Giddings et al. [9] for the production of acetic acid using *Sporomusa ovata*. A membraneless MES was also reported for biomethane production from CO_2_, achieving a generation rate of 4.7 L/ (m^2^∙d) [8].

The second advancement front relates to the development of novel MES electrodes with enhanced properties. According to a recent review [7], the insufficient electrocatalytic ability of electroactive microorganisms is one of the main bottlenecks restricting the further large-scale application of MES. Research on electrodes is constantly advancing, and a plethora of new materials with excellent performance and breakthrough technologies are emerging that can play a vital role in developing MES at a larger scale. In general MES electrodes should be conductive, chemically inert, biocompatible, and with a high surface area to maximize interaction with the methanogenic bacteria. The materials of choice were traditionally metals, carbon-based and, more recently, metal–carbon hybrids. According to recent studies, conductive carbon materials can stimulate direct or indirect interspecies electron transfer (DIET) in co-culture and the anaerobic digestion process by accelerating rates of anaerobic metabolism [11]. In addition, Kim et al. [12] reported that DIET-mediated methanogenesis in MES might be further improved by adding transition metals to carbon electrodes. These metals on the electrode’s surface can serve as highly efficient conduits based on their intrinsic electric properties and catalysts for electromethanogenic reactions. The NPs can improve local electric conductivity while simultaneously reducing the electron transfer activation energy threshold at the surface of the electrodes. Several transition metals and noble catalysts have already being studied such as platinum (Pt), gold (Au), nickel (Ni), palladium (Pd), and iridium (Ir) [12,13,14,15,16,17,18,19,20,21]. Copper (Cu), which also possesses catalytic characteristics and is a lower-cost alternative compared to the abovementioned metals, has not been used in its particle form. Only two bulk-form applications have been detected: Aryal et al. [22] synthesized a cathode by reducing graphene oxide coated on copper foam, and it was used for the microbial electrosynthesis of acetate from CO_2_, whereas Thatikayala and Min [23] developed copper ferrite/reduced graphene oxide nanocomposites using the bio-combustion method. They used this as a cathode catalyst in the microbial reduction of CO_2_ to volatile fatty acids (VFAs) in a single chamber MES.

This study examines the conversion of CO_2_ (as a sole external carbon source) to CH_4_ using membraneless MES inoculated with AGS, where novel carbon cloth (CC) electrodes functionalized with copper nanoparticles (Cu NPs) at two different concentrations were used. The MES was inoculated with anaerobic granular sludge (AGS) to prevent the electrolytic oxygen produced on the anode to inhibit anaerobic microorganisms and especially methanogens. According to Tartakovsky et al. [24], micro-aerobic conditions did not prevent methane production at a laboratory Upflow Anaerobic Sludge Blanket (UASB) treating synthetic wastewater. Since the anaerobic granules are typically larger than 500 μm in diameter and the oxygen penetration depth does not exceed 50 μm, the electrolytic oxygen might be consumed in the outer biofilm layer of granular sludge and does not inhibit methanogens that are positioned in the core of the AGS. The tolerance of methanogens in anaerobic granular sludge to oxygen and their coexistence with facultative bacteria were also previously reported by Kato et al. [25,26]. The novel CC and CC-Cu NPs electrodes synthesized and used within this study were characterized before and after use, and the microbial profile in the cathode and anode biofilm in the AGS was examined at the end of the experiment.

## 2. Materials and Methods

### 2.1. Inoculum

AGS was used as inoculum collected from a full-scale Internal Circulation bioreactor treating dairy wastewater at pH 7.0–7.5 (Charalambides Christis Ltd., Limassol, Cyprus). Total and volatile solids were determined to be 5.7% (wet basis) and 85.4% (dry basis), respectively [27]. Before its use, AGS was sieved (1 mm), washed with distilled water and sealed into a glass bottle (1 L) in a shaking incubator (WiseCube, fuzzy control system, Wisd.23 Laboratory instruments) at 33 °C and 100 rpm for three days.

### 2.2. Electrodes Synthesis and Characterization

CC fabrics were purchased from the Zoltek corporation, having a plain (grid-like) weave pattern, 115 g/m^2^ areal weight, 400 μm thickness and 0.0015 ohm cm electrical resistivity. Rectangular cuts from the CC fabrics were mounted on iron rods to form the electrodes. The plain CC electrodes utilized the cloth material as received, without any further modification. The deposition of Cu NPs was performed in a modified physical vapor deposition system from Mantis Deposition, based on the Nanogen50 nanoparticle source, which is described in details in [28]. The system consists of a 2 inch magnetron sputtering operating in an aggregation zone of high inert gas pressure (argon). By sputtering, atoms are ejected from the metal target and by collisions in the gas phase form NPs. The beam of NPs is introduced to the inline mass filter MesoQ (Mantis deposition) and the distribution of NPs’ diameter is detected in real time during deposition. The varying parameter was the time which controls the coverage of NPs on the CC.

The coverage of Cu NPs on the surface of the samples was examined with atomic force microscopy (AFM, NTEGRA Prima, NT-MDT) using silicon tips in tapping mode. The features of plain electrodes and their condition after use in MES reactors were investigated using a scanning electron microscope (SEM, Quanta, FEI) at various locations on the electrodes. The samples prior to examination were coated with a few nanometers of Au to increase surface conductivity and limit charging effects. Images were acquired at 20 kV accelerating voltage and at multiple magnifications. The built-in energy dispersive X-ray spectroscopy (EDS) system was also employed to probe the elemental composition of the electrodes before and after use in the MES reactors. Raman spectroscopy (B&WTek Raman Microscope) was used to evaluate the vibrational characteristics of the CC fiber network. The spectra were acquired with a 100× objective lens, using a 30 mW 532 nm laser and 120 sec integration time.

X-ray diffraction (XRD) has been employed to characterize the CC electrodes and identify any crystalline phases, formed on the surface of the electrodes after usage in the MES reactors. The electrodes were cut into appropriate small pieces and placed on a low background glass sample holder without any other treatment. The XRD patterns were acquired in an Ultima IV diffractometer, using a Cu tube operated at 40 kV and 40 mA. The incident X-ray beam was configured in parallel mode and partially monochromatized (Cu Ka, λ = 0.154 nm) by a parabolic multilayer mirror. All patterns were collected in the 15–60° 2 theta angular range using the same conditions, with 0.02° step and 1°/min scan speed.

### 2.3. MES System Set-Up

The MES system was developed in a 165 mL serum bottle and a working volume of 120 mL medium was used [29] as well as 10 g NaHCO_3_/L. Forty grams per liter of anaerobic granular concentration was used as inoculum in all the MES-serum bottles. Three different types of electrodes were used: CC, CC functionalized with Cu NPs deposited for 15 min (CC-15minCu) and CC functionalized with Cu NPs deposited for 45 min (CC-45minCu). Each electrode type was tested under voltage (MES) and no voltage conditions to investigate the effect of conductive electrode material on methanogenesis. In addition, serum bottles that contained AGS with no electrodes served as a control (Table 1). The serum bottles were operated for six cycles for 144 days, as shown in Table 2. At the beginning of each cycle, the MES were flushed with CO_2_ (for 5 min), and 30 mL were replaced with concentrated NaHCO_3_/L; so the initial concentration of NaHCO_3_ in the serum bottle was set at 10 g/L (Table 1).

The external potential was applied using a DC power supply (GWInstek, Taiwan). The output voltage in reactors MES-serum bottle was recorded every 5 min by a data acquisition system (Keithley 2700) connected to a computer.

### 2.4. Analytical Methods

Determination of total solids (TS), volatile solids (VS) and sCOD was performed according to Standard Methods (APHA 2005). Regarding sCOD measurement, liquid samples were regularly taken during the experiments, centrifuged at 13,500 rpm for 10 min and after filtration (0.2 μm FLL/MLL acrylic membrane, GVS, Italy) were analyzed using the closed reflux colorimetric method.

Gas composition (H_2_, O_2_, N_2_, CH_4_, and CO_2_ concentrations) analysis was performed using an Agilent Technologies Gas Chromatographer 7820A connected to a Thermal Conductivity Detector (Wilmington, DE) according to Vardanyan et al. [30]. A sample volume of 1 mL was withdrawn from the headspace and injected in GC-TCD. The separation of the gases was achieved using a ShinCarbon ST packed column (Restek Corporation, Bellefonte, PA, USA) using argon (1 mL min^−1^) as a carrier gas. The initial column temperature was held for 1.5 min at 60 °C, then was raised to a final temperature of 155 °C at a rate of 48 °C min^−1^ and maintained for 0.7 min. The injector and detector were maintained at 125 °C and 250 °C, respectively.

VFAs (acetic, propionic, iso-butyric, butyric and valeric acid) were analyzed by a high-performance liquid chromatography (LC-20AD, Shimadzu, Japan) associated with a Shimadzu SPD20 A UV/VIS detector and a Shimadzu SIL-20 A HT auto sampler. Elution was performed isocratically on a Rezex™ ROA-Organic Acid H+ (8%) (150 × 7.8 mm, Phenomenex) with 5 mM H_2_SO_4_ at 50 °C as decribed by Vyrides and Stuckey [31]. The injection volume was 1 μL and the flow rate 0.7 mL min^−1^. The UV detector was set at 210 nm during the analysis.

The energy efficiency (η, %) was used to describe the relationship between the input and output energy in the system, defined as the ratio of the latent heat potential to methane production to the input electrical energy. The energy efficiency was calculated according to:

(1) $$n=\frac{\left(V_{\mathrm{CH}4}\right)\times 39.829}{U\;\int_{0}^{t}I.\mathrm{dt}}$$

where V_CH4_ is the cumulative methane production (L CH_4_) at time t (s), U is the input voltage (V), I is the current (A), and 39.829 kJ L^−1^ is the energy content based on Gibbs’ free energy of CH_4_ oxidation (−890.31 kJ mol^−1^) [32].

All analytical measurements were performed in duplicate. The absolute difference between the average and each measurement was less than 10%; otherwise, the analysis was repeated.

### 2.5. High-Throughput 16S rRNA Gene Sequencing

At the end of all experiments, samples up to 250 mg were collected from all the serum bottles. Specifically, the MES-serum bottles samples were collected from the anode and cathode biofilm as well as from the AGS in the bottom. A DNeasy PowerSoil Pro kit (Quiagen, Germany) was used for the total genomic DNA extraction following manufacturers’ instructions and the final extracts were analyzed by DNASense Apps Company (Denmark) as described by Gatidou et al. [33].

## 3. Results and Discussion

### 3.1. CC Electrodes Functionalized with Cu NPs

**Figure 1:**
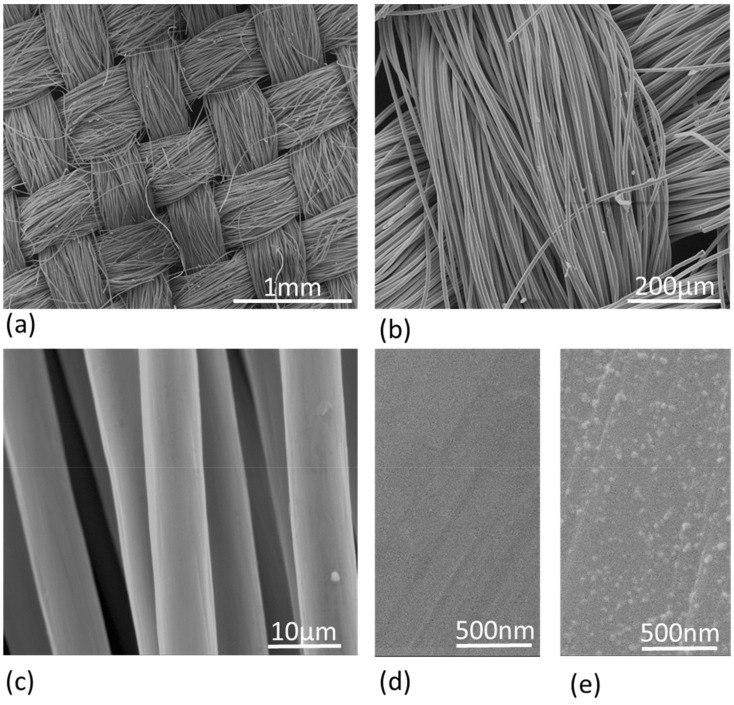
SEM images of a plain CC fabric at low (**a**,**b**), medium (**c**) and high (**d**) magnification. High magnification image of CC-Cu showing the copper nanoparticles (**e**).

Figure 1 shows SEM images at various magnifications of the plain CC (a-d) used for the production of electrodes utilized in the MES systems developed within this study. The 90 degrees weaved structure is evident, generated from micron scale fibers, providing a robust material with a significant surface area for interaction with the microorganisms of the AGS in the MES system. The copper nanoparticles deposited in the case of CC-Cu electrodes are shown in the high magnification image (×100,000) in Figure 1e. More precise geometrical characteristics of the deposited Cu NPs are quantified through AFM measurements.

**Figure 2:**
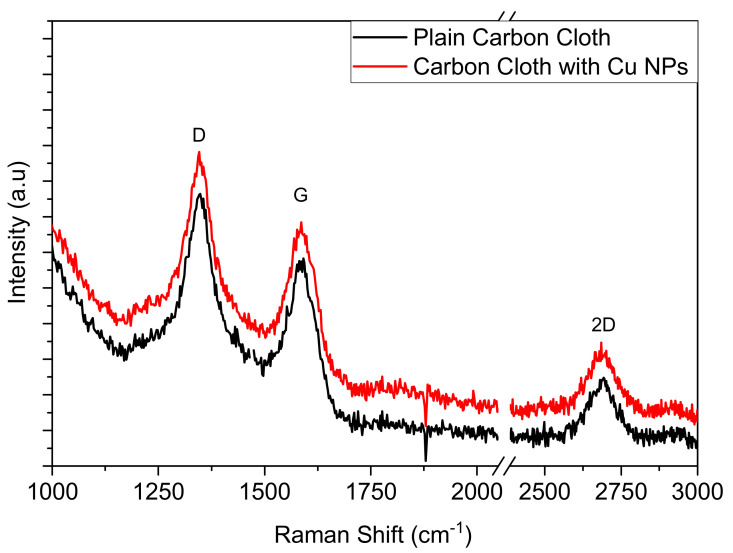
Raman spectrum of plain CC fabric and decorated with Cu NPs, exhibiting the characteristic peaks D, G and 2D of carbon fibers.

The atomic structure of this material was probed using a Raman Microscope which is an important tool for characterizing carbon allotropes. The spectra for plain CC are shown in Figure 2 with three characteristic peak intensities at 1335 cm^−1^, 1580 cm^−1^, and 2700 cm^−1^ that correlate with the D, G and 2D bands of carbon respectively. The D band relates to the disorder of carbon materials, while the G band is primarily an in-plane vibrational mode of sp^2^ hybridization states, whereas the 2D band is an overtone of the D band, indicative of long range order. A D band intensity greater than the G band suggests a material with disordered dominance which relates to a moderate mechanical response and moderate electrical conductance carbon, consistent with the manufacturer’s specifications. Consistent with the literature the metallic nanoparticles do not introduce any additional peaks in the Raman spectra.

**Figure 3:**
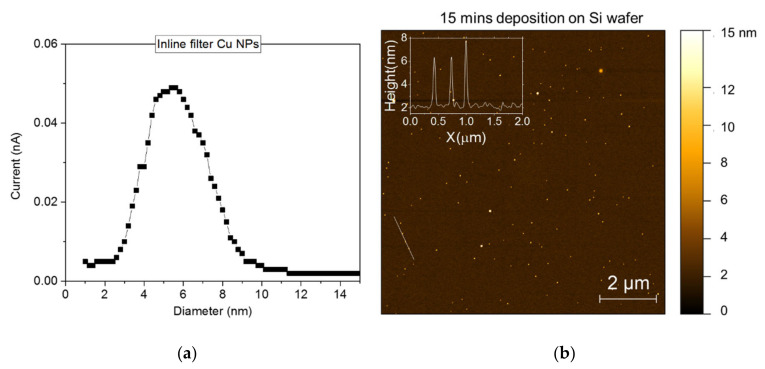
(**a**) MesoQ filter grid detection current versus nanoparticle diameter and (**b**) AFM 10 × 10 microns scan of a Si wafer piece deposited with Cu NPs (15 min) along with the CC samples. The inset graph shows the line height profile of the white line located on the lower left side of the AFM scan, dissecting 3 NPs.

The CC fabrics were functionalized with Cu NPs in an attempt to enhance the interaction between the microorganisms and the electrodes during the MES process. NPs deposited using the Nanogen source had a Gaussian particle size distribution around a mean of 6 nm in diameter, as measured with the MESOQ filter and presented in Figure 3a. In order to ensure the proper production and deposition of NPs, AFM images of NPs deposited on a silicon wafer were collected and analyzed. Figure 3b shows an indicative AFM of Cu NPs deposited for 15 min with a cross sectional representation (inset) verifying that the NPs diameters are primarily in the range of 5–8 nanometers.

**Figure 4:**
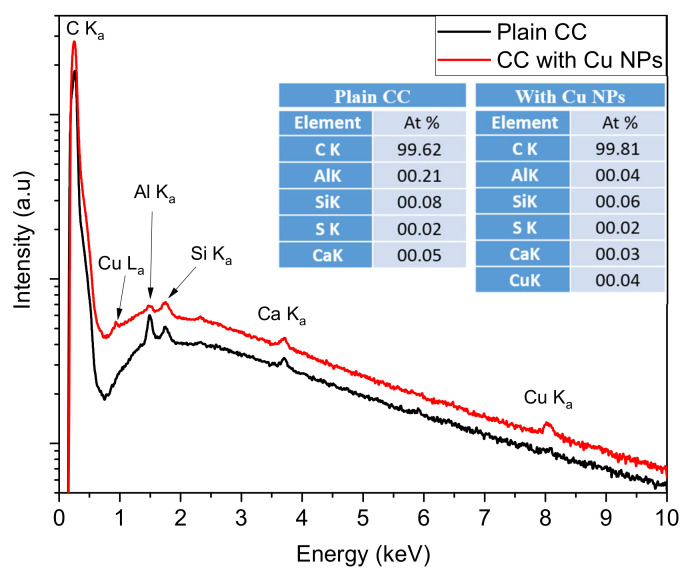
EDS spectra of plain CC (black line) and CC coated with Cu NPs (red line). The inset tables show the corresponding elemental atomic composition of these spectra.

To ensure the proper deposition and effective functionalization of the CC electrodes with Cu NPs, plain CC and CC decorated with Cu NPs (CC-45minsCu) were probed using an energy dispersive X-ray spectrometer. Figure 4 shows the X-ray spectra of the two materials that verify the existence of Cu NPs in the latter case, where Cu peaks are detected in their respective characteristic energies with an estimated atomic percentage of copper on the order of 0.04 at.%. Beyond carbon and copper one can see traces of Si, S and Ca, which are probably residuals of the CC material. Al is related to the stubs used for sample holders.

### 3.2. MES System Performance

**Figure 5:**
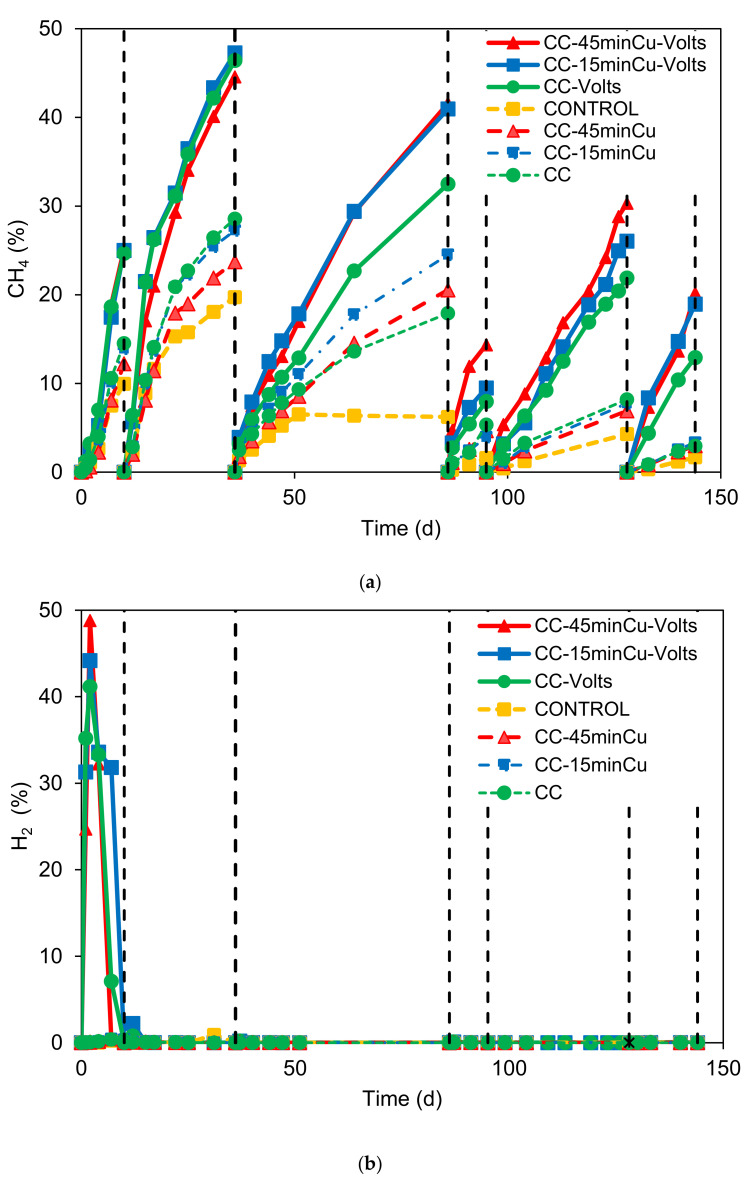
(**a**) Methane composition over time. (**b**) H_2_ composition over time.

Figure 5a shows the methane production (%) over time at the different serum bottles. During the experiment (six cycles), the methane was consistently higher at the serum bottles with CC electrodes with applied voltage. The highest CH_4_ (around 46%) was found at the second cycle after 16 days. However, the system’s performance declined during the following cycles, nevertheless the CH_4_ % composition was almost doubled compared to the serum bottles without voltage. The CC electrodes with Cu NPs and voltage exhibited higher methane production than plain CC with voltage for cycles three to six. At the first two cycles, no significant difference was identified for these electrodes; however, their performance was higher than that of the samples without voltage. The serum bottles with CC electrodes (without voltage) showed a higher performance than the control. This is in line with the study of Feng et al. [34], that showed that the addition of conductive carbon cloth is a feasible strategy to enhance AD performance through the stimulation of DIET in a mixed culture. However, solid evidence for microorganisms participating in DIET is lacking, and the mechanism of the shift from IHT to DIET remains unknown [34].

The H_2_ composition over time is presented in Figure 5b. During the first cycle, the serum bottles with electrodes and voltage generated a significant amount of H_2_ (41–48%). At the beginning of the second cycle, the serum bottle with the voltage generated around 2.5%. At the following days until the end of the experiment, the H_2_ was consistently lower than 1%. During the first cycles, it is likely that the cathode potential is low enough to produce water reduction from the electrodes. In addition, at this stage the biofilm was likely not yet developed on the surface of the electrode, resulting in the accumulation of H_2_. Apart from this, during the first cycles it is likely that the iron rods of the CC electrodes were oxidized and generated H_2_. Subsequently, the H_2_ was utilized by hydrogenotrophic methanogens and homoacetogens to produce methane and acetic acid, respectively. During the first cycle, the rate of H_2_ production was likely higher than the rate of H_2_ utilization by hydrogenotrophic microorganisms. However, in the following cycle, the hydrogenotrophic methanogens were enriched and the hydrogen production rate declined, and as a result, no substantial hydrogen was identified. Therefore, the high methane in the first cycles it is likely due to abiotic production of H_2_ followed by its utilization by hydrogenotrophic microorganisms. After the first cycle, siderite was created on the surface of iron rod, and this probably blocked further oxidation of iron and subsequent hydrogen generation. However, after the first cycles, despite the increase in the voltage, the oxidized products have not contributed further to H_2_ production. The high percentage of hydrogenotrophic methanogens in all the samples from MES system is in line with the findings from this section.

**Figure 6:**
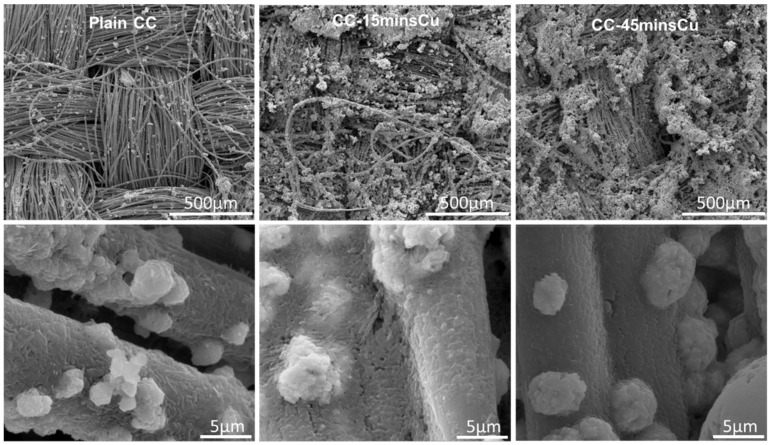
SEM images of low (**top row**) and high (**bottom row**) magnification of used plain CC (**left**), CC-15minCu (mid) and CC-45minCu (**right**) electrodes.

**Figure 7:**
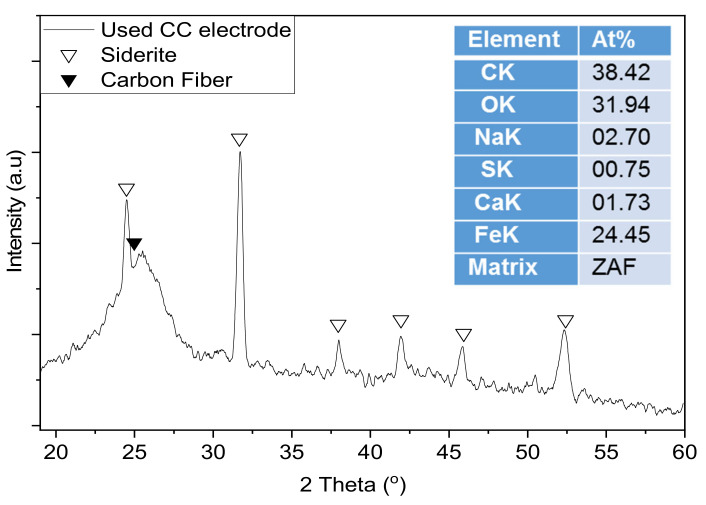
XRD pattern of a used electrode. Siderite and carbon fiber peaks are marked with a white and black triangle, respectively. The inset table shows the elemental atomic composition of a wide area of the used CC electrode.

In order to better investigate the interaction between microorganisms and electrodes SEM and XRD investigations were performed on the extracted electrodes after they have been used in the MES systems. Figure 6 shows SEM images at two different magnifications for all electrodes used in this study after ~5 months of operation in the MES systems. It is evident that as the Cu NPs content increases on the CC surface the microbial/deposition layer increases, suggesting a more favorable interaction in the presence of Cu NPs. Various microbes attached on the CC surface can be observed at higher magnifications. Furthermore, in order to detect any crystalline phases that probably exist on the surfaces of those materials, we performed XRD. Figure 7 shows an indicative diffraction pattern (we have observed similar trends for all electrodes) after the electrodes were used in the MES system and in interaction with the anaerobic microbes suggesting that the dominant biocorrosion crystalline product of the process is the iron carbonate phase of siderite (FeCO_3_). The availability of carbon is a result of the metabolic pathway of converting CO_2_ to CH_4_ and part of it appears to be chemically forming siderite.

### 3.3. VFAs

**Figure 8:**
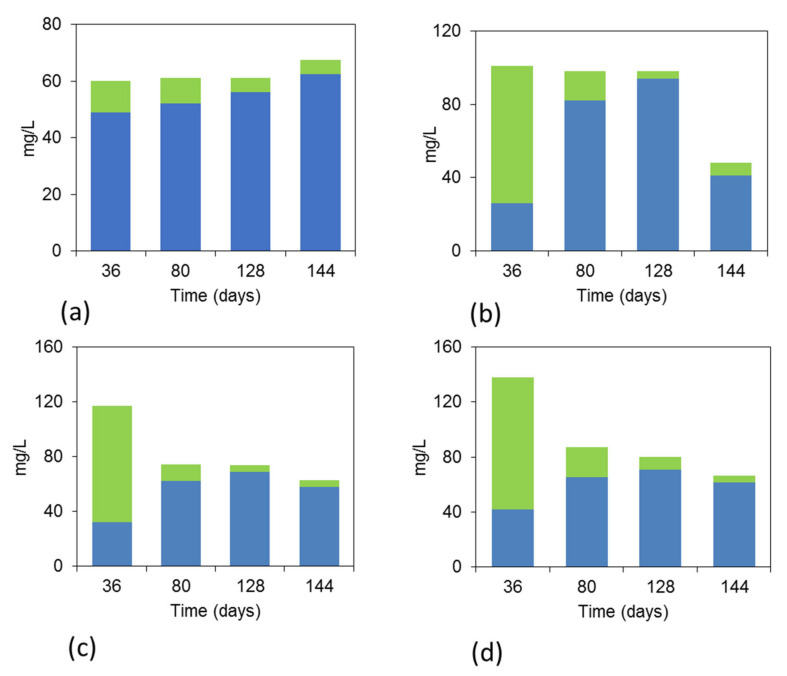
Variation in VFAs composition (mg L^−1^) over time in the different serum bottles for the (**a**) Control, (**b**) CC-Volts, (**c**) CC-Cu15mins-Volts and (**d**) CC-Cu45mins-Volts; blue represents the acetic acid and green the propionic acid.

As shown in Figure 8a–d, in the first cycle on day 36, the serum bottles exposed to the voltage generated a higher concentration of volatile fatty acids than the other samples. On day 36, the serum bottles exposed to voltage has a higher propionic acid concentration that than the acetic acid and this could be due to the relatively high hydrogen that was produced on the first cycle. It is likely at these conditions, the rate of hydrogen production from the electrolysis was higher than the rate of hydrogen utilization by hydrogenotrophic methanogens or homoacetogens, and this has not facilitated the propionic acid biodegradation that requires low hydrogen partial pressure [27]. In the next cycles, the acetic acid was the dominant VFAs; however, its concentration was below 100 mg L^−1^ probably due to its utilization by acetoclastic methanogens or due to the uptake of H_2_ at a faster rate by hydrogenotrophic methanogens than the homoacetogens.

### 3.4. Energy Efficiency

**Figure 9:**
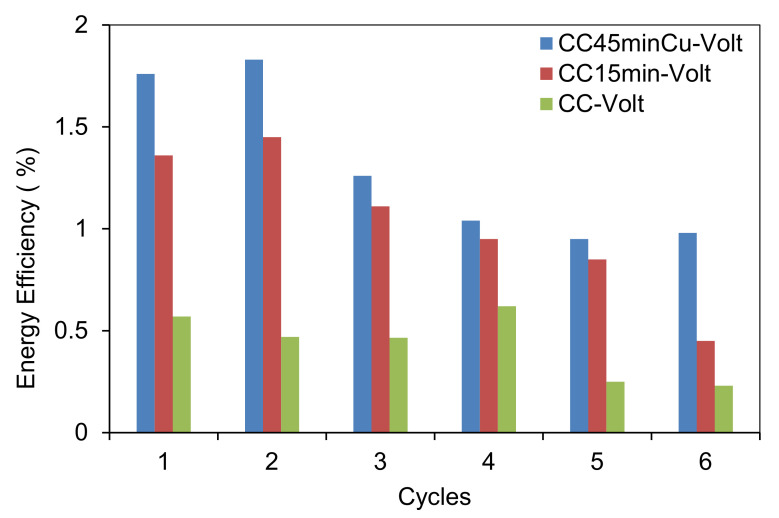
Energy Efficiency for serum bottles CC-volts, CC15min-volts and CC45minCu-volts over the 6 cycles.

The average current density in each cycle and the energy efficiency of the MES system are pointed out in Table 3 and in Figure 9. As shown in Table 3, the current for the serum bottle with copper electrodes (CC-45minCu-Volt) is relatively constant, despite the lower current in this higher methane production. This is in line with the higher energy efficiency in this system. The same trend but with a higher fluctuation in current was found for the serum bottle (CC-15min-Volt). The serum bottle with CC-Volt had relatively higher current density compared with the other serum bottles, especially in the first two cycles; however, the produced CH_4_ was not high probably due to higher energy losses. This is an indication that the main mechanism for methane production is through hydrogen production and not through DIET, this is in line with the microbial findings that the hydrogenotrophic methanogens was the dominant genus. However, more research can be conducted to elucidate the mechanism in this system.

The energy efficiency of the MES system is pointed out in Figure 9. The efficiency was relatively low for all conditions, less than 2%, more likely due to the absence of proton exchange membrane and the high internal resistance of the system. However, the efficiency of serum bottles with CC and Cu NPs was higher than the serum bottles with CC only. For serum bottles the energy recovery efficiency decreased with increasing input voltage from 1 to 2 V. According to Wang et al. [32] increasing the applied voltage, although it was able to increase the current density, increased the internal resistance of the system, which resulted in increased energy loss. Yuan et al. [35] pointed out that increased CH_4_ production in an MEC-AD reactor would decrease the electric energy consumption around 20-fold, while the economic benefits of increased CH_4_ production could fully cover the input power cost.

### 3.5. Microbial Profile Analysis

**Figure 10:**
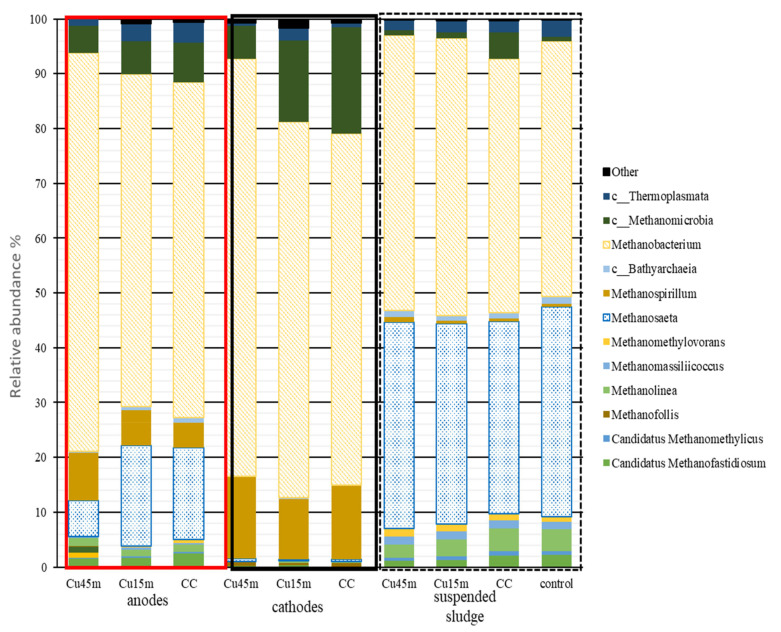
Archaea relative abundance % at a genus level. Genus had a relative abundance <0.5% were assigned to “others”. Sample nomenclature: Cu45min: Carbon cloth deposited with copper nanoparticles for 45 min, Cu15min: Carbon cloth electrode deposited with copper nanoparticles for 15 min, CC: carbon cloth electrode, Control: serum bottle inoculated with anaerobic granular sludge no electrodes.

The relative microbial abundance of archaea for the three anodes, cathodes and suspended biomass is shown in Figure 10. The *Methanobacterium* was the most dominant genus in all samples, and it is found in higher abundance on the cathodes, then on the anodes, and then in the suspended biomass. *Methanospirillum* was the second most abundant genus and is following the same trend as *Methanobacterium*—high relative abundance in the cathodes, lower abundance on the anodes and was slightly present in the suspended sludge. *Methanobacterium* and *Methanospirillum* are hydrogenotrophic methanogens and utilize the hydrogen produced in the cathodes. The results align with Siegert et al. [36], who reported cathodes’ attachment mainly by the genus *Methanobacterium* while studying methane production in acetate-fed MECs systems. In addition, Gatidou et al. [33] and Li et al. [37] found a high relative abundance for *Methanobacterium* on the electrodes at a microbial electrolysis cell compared to the control. On the other hand, *Methanosaeta* (obligate acetoclastic methanogens) was most dominant in the suspended sludge (35–39%), followed by high abundance in the anode (6–18%) and was little present in the suspended sludge. *Methanosaeta* is a prominent microbial group responsible for methanogenic granule formation [38]. Previous work with MECs using iron rod electrodes also revealed a high abundance of *Methanosaeta* in suspended sludge compared to the electrode surface [33]. Based on these findings a tentative conclusion can be stated that methane is mainly generated by hydrogenotrophic methanogens from the electrodes and through acetoclastic methanogens from the suspended AGS.

**Figure 11:**
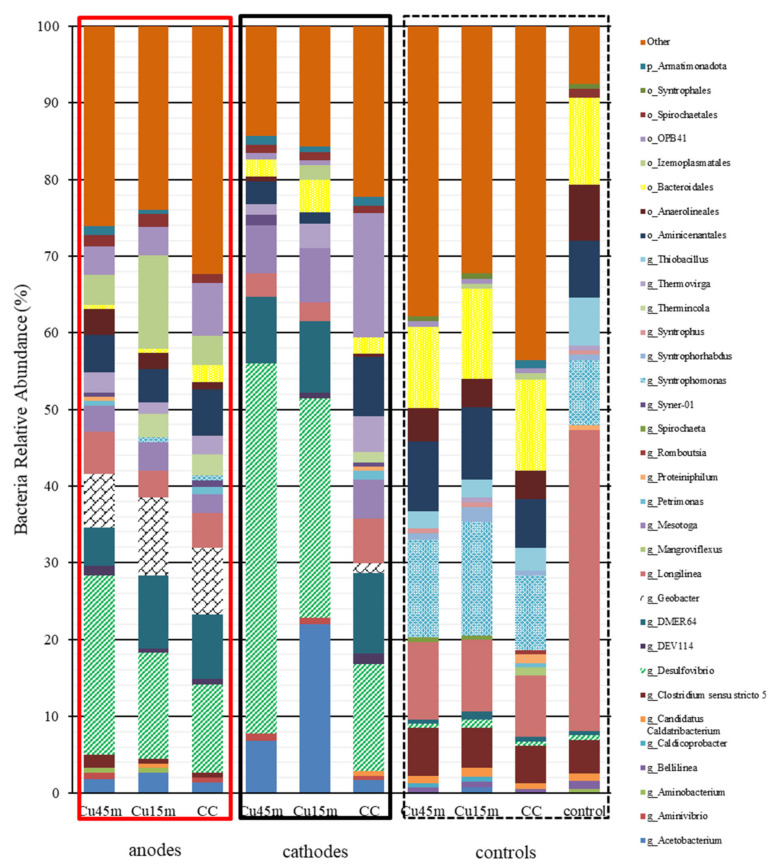
Bacteria relative abundance % at a genus level. Genus had a relative abundance <0.5% were assigned to “others”. Sample nomenclature: Cu45min: Carbon cloth deposited with copper nanoparticles for 45 min, Cu15min: Carbon cloth electrode deposited with copper nanoparticles for 15 min, CC: carbon cloth electrode, Control: serum bottle inoculated with anaerobic granular sludge no electrodes.

The microbial profile of bacteria at the genus level can be seen in Figure 11. The genus *Geobacter* was identified only in the anodes regarding relative bacterial abundance at around 6–10%. *Geobacter* is an electroactive bacterium; previous research found that *Geobacter* dominated the anode microbial community [32,39]. *Geobacter* performs DIET, which has a higher electron transfer efficiency than anaerobic digestion’s electronic respiratory transfer chain [32]. *Desulfovibrio* was the most dominant genus in the cathodes; however, its relative abundance was significantly higher for the cathodes with Cu NPs. The same trend but with a lower relative abundance than the cathodes electrodes was found for *Desulfovibrio* on the anodes. According to [40], several microorganisms within the deltaproteobacteria class have been proved to possess electroactivity. This genus was also identified on iron rod electrodes during MEC treating bilge water [33]. DEMR64 (family *Rikenellaceae*, *Phylum Bacteroidota*) and *Mesotoga* was identified at a high relative abundance on the anode and cathode but not in the suspended sludge. *Mesotoga* bacteria could utilize acetate and it Li et al. [37] also found an increase of *Mesotoga* in MEC when potential was applied. A homoacetogenic bacteria (*Acetobacterium*) was identified in relatively high abundance only in the cathodes with Cu NPs, more likely due to the higher concentration of hydrogen produced on these cathodes. On the other hand, the genus *Clostridium sensu stricto*, *Syntrophomonas* and *Thiobacillus* were mostly found in suspended sludge and were slightly identified in the electrodes. *Clostridium* could reduce CO_2_ to produce acetic acid, which might be responsible for the relatively high propionic acid [32]. *Clostridium* has been identified as acetogenic bacteria with bioelectrochemical activity, and it could oxidize acetate to produce H_2_/CO_2_ and grow symbiotic ally with H2 consuming methanogens [41]. *Longilinea* was identified in all samples; however, it had its higher abundance in 39.2% in the suspended biomass in control. Zhang et al. [13] also found *Longilinea* on an anode in a microbial electrolysis system treating wastewater.

## 4. Conclusions

This study investigated the conversion of CO_2_ (as a sole external carbon source) to CH_4_ using membraneless MES inoculated with AGS. Three types of electrodes were independently tested: CC and CC electrodes functionalized with Cu NPs deposited for 15 min and 45 min, respectively.

The experiment took place in six cycles for a total of 144 days. The methane was around 2.5 times higher in the serum bottles with CC electrodes with voltage than in serum bottles without voltage. The highest CH_4_ (around 46%) was found in the second cycle after 16 days. However, the system’s performance declined during the following cycles probably because of low hydrogen generated from the abiotic process and therefore low methane. The MES with CC electrodes containing Cu NPs exhibited a higher performance than the MES with plain CC electrodes. This is likely due to H_2_ generation by electrodes that contain Cu NPs and because of the higher electrical conductivity of those electrodes that probably facilitate the microbial reactions. This difference was more pronounced after the second cycle.

During the first cycle, the serum bottles with electrodes and voltage generated a significant amount of H_2_ (41–48%). However, in the following cycles, the H_2_ was significantly lower.

Acetic acid was mainly detected in the first cycle, whereas in the last cycles, propionic acid was the primary carboxylic acid identified in all serum bottles.

The efficiency was relatively low for all conditions. However, the efficiency of serum bottles with CC and Cu NPs was higher than for the serum bottles with CC only. The energy recovery efficiency decreased with increasing input voltage from 1 to 2 V for serum bottles.

*Methanobacterium* was the most dominant genus in all samples, and it was detected in higher abundance on the cathodes, then on the anodes, and then in the suspended biomass. The genus *Geobacter* was identified only in the anodes regarding relative bacterial abundance at around 6–10%. *Desulfovibrio* was the most dominant genus in the cathodes; however, its relative abundance was significantly higher for the cathodes with Cu NPs.

## Figures and Tables

**Table 1:** Parameters used in each serum bottle.

CODE	Serum Bottle
CC-45minCu-Volts	Developed electrode that copper nanoparticles were deposited for 45 min. A voltage was applied.
CC-15minCu-Volts	Developed electrode that copper nanoparticles were deposited for 15 min. A voltage was applied.
CC-Volts	Commercial carbon cloth
CC-45minCu	Developed electrodes used in serum bottle without applied external potential
CC-15minCu	Developed electrodes used in serum bottle without applied external potential
CC	Developed electrodes used in serum bottle without applied external potential
Control	Only anaerobic granular sludge no electrodes

**Table 2:** Operational conditions and duration of each cycle.

Cycle	Days	Volts *	Carbon Source
1st cycle	1–10	1	CO_2_ + 10 g NaHCO_3_/L
2nd cycle	10–36	1	CO_2_ + 10 g NaHCO_3_/L
3rd cycle	36–86	1	CO_2_ + 10 g NaHCO_3_/L
4th cycle	86–95	1	CO_2_ + 10 g NaHCO_3_/L
5th cycle	95–128	2	CO_2_ + 10 g NaHCO_3_/L
6th cycle	128–144	2	CO_2_ + 10 g NaHCO_3_/L

* External potential was applied only to the serum bottles as shown at Table 1.

**Table 3:** Average current density for serum bottles CC-volts, CC15min-volts and CC45minCu-volts over the 6 cycles.

Average Current Density (mA)						
Cycle	1st	2nd	3rd	4th	5th	6th
CC-45minCu-Volt	5.2	5.1	1.8	5.5	3.3	4.9
CC-15minCu-Volt	6.4	7.5	2.1	4.1	3.1	11.2
CC-Volt	13.5	20.2	4.2	6	7.5	11

## Data Availability

Data can be available upon request from the authors.
